# Romosozumab and Blosozumab: Alternative Drugs of Mechanical Strain-Related Stimulus Toward a Cure for Osteoporosis

**DOI:** 10.3389/fendo.2015.00054

**Published:** 2015-04-21

**Authors:** Toshihiro Sugiyama, Tetsuya Torio, Tsuyoshi Miyajima, Yoon Taek Kim, Hiromi Oda

**Affiliations:** ^1^Department of Orthopaedic Surgery, Saitama Medical University, Saitama, Japan

**Keywords:** osteoporosis, mechanostat, sclerostin, romosozumab, blosozumab

## Treat-to-Target Strategy in Osteoporosis

In addition to other chronic diseases such as hypertension, hypercholesterolemia, and diabetes, a treat-to-target strategy was recently applied in rheumatoid arthritis and has now been discussed in osteoporosis. An important goal of osteoporosis therapy is normal risk of hip fracture associated with significant morbidity and mortality, but the anti-fracture efficacies of currently approved drugs are limited (1, 2). Although fundamental methods to effectively prevent osteoporotic fracture include pharmacological treatment of sarcopenia that results in improving bone fragility as well as reducing fall risk, the present article focuses on anti-sclerostin antibodies such as romosozumab and blosozumab, the investigational agents for osteoporosis, and provides new insights into their effects from natural homeostatic system in the skeleton.

## Alternative Drugs of Mechanical Strain-Related Stimulus

**Figure 1:**
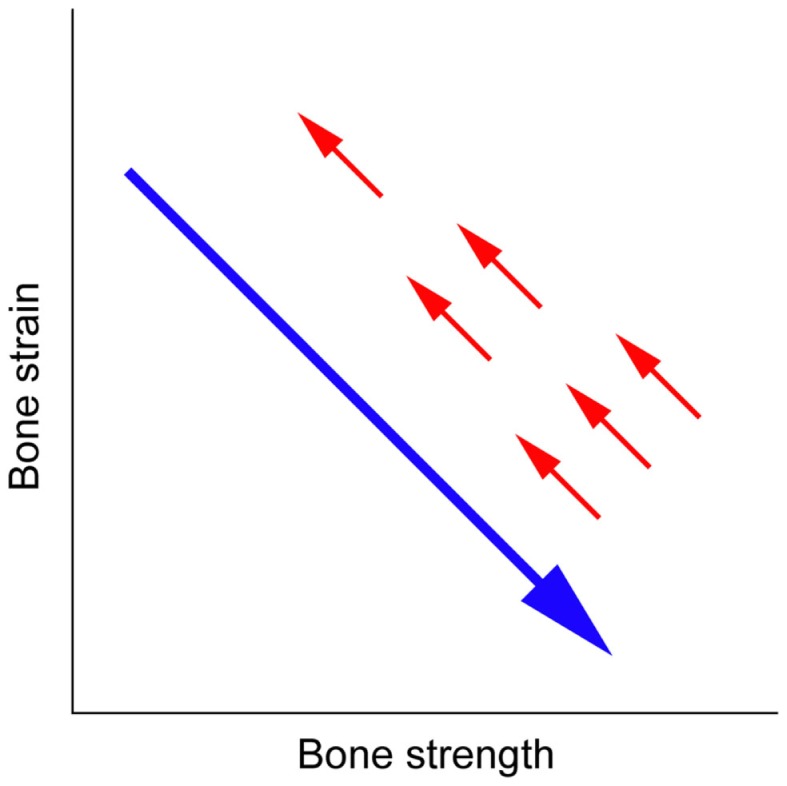
**Mechanical strain-related feedback control of bone strength**. A long arrow indicates the effect of osteoporosis therapy that increases bone strength and thus decreases bone strain from physical activity, and short arrows indicate the negative feedback control of bone strength that returns bone strain to its pre-treatment level (5).

The human skeleton normally responds to the change in local mechanical environment at each skeletal site to maintain resultant elastic deformation (strain) of bone; increased or decreased bone strain would induce bone gain or loss, respectively (3–5). This mechanical strain-related feedback control called the mechanostat (6, 7) plays a key role in the management of osteoporosis; increased bone strength by an osteoporosis drug results in decreased bone strain, indicating that the effect of osteoporosis therapy is limited by the mechanostat (Figure 1) (5). Approaches to reduce the limitation include pharmacologically enhancing skeletal response to mechanical stimulation (8), but this might not efficiently reduce the risk of fall-related hip fracture because the skeleton is adapted to the mechanical environment resulting from habitual physical activity but not to the unusual direction of mechanical force by falls. Consequently, an ideal strategy is to develop an alternative agent of mechanical strain-related stimulus (5).

One example would be investigational anti-sclerostin antibodies such as romosozumab and blosozumab; experimental evidence has established that the production of sclerostin secreted by osteocytes is increased by skeletal disuse and decreased by skeletal loading (9, 10). In addition, bone formation induced by intermittent treatment with parathyroid hormone is associated with the inhibition of sclerostin (11), suggesting that teriparatide could partly have a similar effect. There is, however, an obvious difference between the effects of anti-sclerostin antibodies and teriparatide, if injected daily (8), on bone remodeling; remodeling-based, coupled bone resorption and formation are significantly promoted by the latter, but not by the former.

## Modeling-Based Effects of Anti-Sclerostin Antibodies

In contrast to bone remodeling, modeling-based bone formation and resorption are not coupled, and mechanical stimulation is a natural uncoupling factor that stimulates bone formation and inhibits bone resorption; experimental data in skeletally mature animals (12, 13) show that strong suppression of bone resorption by risedronate or denosumab does not impair modeling-based bone formation induced by artificial mechanical loading or habitual physical activity. In agreement with the above suggestion that investigational anti-sclerostin antibodies such as romosozumab and blosozumab are alternative drugs of mechanical strain-related stimulus, an experiment using male cynomolgus monkeys found a marked increase in modeling-based bone formation by romosozumab (14) and phase 2 clinical studies in postmenopausal women confirmed that both romosozumab and blosozumab treatments rapidly induced an increase in bone formation and a decrease in bone resorption (15, 16).

## Optimal Doses of Anti-Sclerostin Antibodies

In postmenopausal women with low areal bone mineral density (BMD), romosozumab and blosozumab treatments for 1 year increased areal BMD at the lumbar spine and hip dose-dependently; mean changes from baseline in areal BMD at the lumbar spine, total hip and femoral neck by romosozumab [placebo vs. highest dose (210 mg every 1 month)] were −0.1 vs. 11.3%, −0.7 vs. 4.1%, and −1.1 vs. 3.7%, respectively, while those by blosozumab [placebo vs. highest dose (270 mg every 2 weeks)] were −1.6 vs. 17.7%, −0.7 vs. 6.7%, and −0.6 vs. 6.3%, respectively (15, 16). Notably, however, areal BMD at the one-third radius was not changed by the highest dose of romosozumab (−0.9 vs. −1.2%, respectively) and non-significantly increased only by the highest dose of blosozumab (−1.4 vs. 0.9%, respectively) (15, 16).

The effects of anti-sclerostin antibodies at the radius appear to reflect the fact that forearm is not exposed to high levels of mechanical strain under normal physical activity. Experimental evidence that the production of sclerostin secreted by osteocytes is increased by skeletal disuse and decreased by skeletal loading (9, 10) suggests that the levels of sclerostin expression in non-weight-bearing bones such as the radius could be higher than those in weight-bearing bones such as the lumbar spine and hip. Consequently, it would be possible to speculate that even highest doses of romosozumab and blosozumab selected in phase 2 clinical studies were not enough for the radius. Indeed, the strongest effects on areal BMD at the lumbar spine and hip were achieved with the highest dose of blosozumab and only this regimen resulted in a trend of increase in areal BMD at the radius (15, 16); further higher doses of blosozumab might increase areal BMD at the radius dose-dependently. Several lines of evidence to support this hypothesis include (i) patients with sclerosteosis due to deficiency of sclerostin have higher areal BMD at the radius as well as the lumbar spine and hip (17) and (ii) appropriate doses of anti-sclerostin antibodies effectively increase bone mass in animals with skeletal disuse or unloading (18, 19).

If the above logic is correct, the highest doses of romosozumab (210 mg every 1 month) and blosozumab (270 mg every 2 weeks) are unlikely to cause unwanted bony overgrowth at non-weight-bearing sites such as the face and skull in postmenopausal women with osteoporosis. In contrast, however, further higher doses of these drugs would be required to improve skeletal fragility in patients with reduced physical activity; one useful indicator to determine optimal doses of anti-sclerostin antibodies could be areal BMD at the radius.

## Limitation of Treatment with Anti-Sclerostin Antibodies

Both romosozumab and blosozumab treatments in postmenopausal women with low areal BMD showed that marked changes in circulating bone formation and resorption markers returned to the pre-treatment levels within a year despite the continued treatments (15, 16). The existence of other mechanotransduction pathways independent of sclerostin (20) indicates that treatment with an anti-sclerostin antibody cannot escape from the mechanostat-related limitation of osteoporosis therapy (5).

The relation between circulating sclerostin and bone mass would support this theory. Sclerostin-related high bone mass in patients with sclerosteosis or van Buchem disease and heterozygous carriers of these diseases is linked to lower levels of circulating sclerostin (21, 22), while circulating sclerostin and bone mass in normal women and men have a positive correlation (23–25). This discrepancy suggests that higher bone mass associated with other mechanotransduction pathways independent of sclerostin would cause lower mechanical strain in the skeleton and thus could result in compensatory higher sclerostin production according to the mechanostat, although the positive correlation between circulating sclerostin and bone mass is also influenced by the fact that higher bone mass results in more osteocytes, which are the main source of sclerostin (26).

## Withdrawal of Treatment with Anti-Sclerostin Antibodies

Results of 1-year post-treatment follow-up after 1-year treatment with blosozumab were recently reported in the phase 2 clinical trial of postmenopausal women with low areal BMD. Mean changes from baseline in areal BMD at the lumbar spine, total hip, and femoral neck by the highest dose (270 mg every 2 weeks) of blosozumab (1-year treatment vs. 1-year treatment plus 1-year follow-up without treatment) were 17.7 vs. 6.9%, 6.7 vs. 3.9%, and 6.3 vs. 5.3%, respectively (27).

The mechanostat indicates that bone strength returns to baseline after the withdrawal of treatment (5) and the speed of this reverse change depends on the drug (28–30). The above site-specific difference in the reduction of areal BMD could partly result from less mechanical loading at the lumbar spine, possibly associated with higher sclerostin production (9, 10). Treatment with an anti-sclerostin antibody can reinforce the fragile skeleton by non-site-specific bone apposition, while its discontinuation would result in mechanical strain-related, site-specific bone loss. Local bone strain from normal physical activity is lower in the inner compartments, suggesting that bone loss caused by the mechanostat-related negative feedback is higher at the trabecular and endosteal surfaces. In contrast, newly formed bone at the periosteal surface through modeling-based apposition might not be resorbed because of a lack of efficient bone resorption in this region.

## Conclusion

Anti-sclerostin antibodies such as romosozumab and blosozumab are the alternative drugs of mechanical strain-related stimulus that can overcome the mechanostat-related limitation of osteoporosis therapy (Figure 1) (5). It is expected that these agents will make a treat-to-target strategy in osteoporosis possible in the near future. Further studies are desired to investigate their optimal doses, especially depending on the levels of habitual physical activity, as well as appropriate duration of the treatments.

## Conflict of Interest Statement

The authors declare that the research was conducted in the absence of any commercial or financial relationships that could be construed as a potential conflict of interest.
